# The Comparison of Pain Caused by Suprapubic Aspiration and Transurethral Catheterization Methods for Sterile Urine Collection in Neonates: A Randomized Controlled Study

**DOI:** 10.1155/2014/946924

**Published:** 2014-01-23

**Authors:** Vajihe Ghaffari, Saeed Fattahi, Mohsen Taheri, Mohammad Khademloo, Roya Farhadi, Maryam Nakhshab

**Affiliations:** ^1^Department of Pediatrics, Faculty of Medicine, Mazandaran University of Medical Sciences, Sari, Mazandaran, Iran; ^2^Department of Community Medicine, Faculty of Medicine, Mazandaran University of Medical Sciences, Sari, Mazandaran, Iran; ^3^Division of Neonatology, Boo-Ali Sina Teaching Hospital, Mazandaran University of Medical Sciences, P.O. Box 48158-38477, Sari, Iran

## Abstract

This study was performed to compare the levels of pain experienced by young infants undergoing either suprapubic aspiration (SPA) or transurethral catheterization (TUC) for the collection of sterile urine samples. This prospective randomized clinical trial was conducted in hospitalized neonates in a university-affiliated hospital. Patients who required urine cultures were randomly assigned into one of two groups, the SPA or TUC group. The infants' faces were videotaped, and the changes in the facial expression and physiological parameters during the procedure were scored using the Premature Infant Pain Profile (PIPP) in a blind manner. The primary outcome was the severity of the pain experienced during each procedure, and the secondary outcomes were the success rate, the duration, and the complications of each procedure. Ninety-four percent of male infants in the TUC group and 77.3% in the SPA group were uncircumcised (*P* = 0.1). The mean (SD) of the PIPP pain scores did not differ between groups (9.95 ± 3.7 in SPA and 9.64 ± 3.2 in TUC, *P* = 0.6). The duration of TUC was longer. Both methods can be used to collect urine from neonates, but the difficulty of performing TUC on females and uncircumcised males should be considered.

## 1. Introduction

Sterile urine samples must be collected from many hospitalized neonates to rule out urinary tract infections [1]. There are two recommended methods for collecting sterile urine samples from neonates, suprapubic aspiration (SPA) and transurethral catheterization (TUC) [2]. SPA is considered the “gold standard” [3] technique for sterile urine collection in neonates [1, 3, 4], but TUC has an acceptable sensitivity of 95% and a specificity of 99% if colony counts greater than 1000 CFU/mL are considered positive [3]. Neither method always successfully collects a sufficient volume of urine [1, 5–7], and both methods are invasive and painful [1, 5].

Neonates can experience pain [1], and experiencing severe pain for a long duration may contribute to neonatal morbidity [8]. Subjection to repeated painful stimuli during the neonatal period may lead to exaggerated responses to pain in later childhood [1, 8]. The “prevention and management of pain and stress” [8] is regarded as a necessary strategy in the care of neonates [8].

To our knowledge, only two studies have compared the pain experienced by neonates during these two procedures [1, 5]. In one study, which was conducted on infants less than two months old, the authors concluded that SPA may be more painful for circumcised boys and for girls [1]. A similar conclusion was reached in the other study, for which the study population was premature neonates [5]. The purpose of the present study was to compare the severity of pain experienced by neonates during SPA and TUC. We compared the success rates and early complications of these two procedures as our secondary objectives.

## 2. Patients and Methods

This study was conducted between January 2011 and September 2011. This study was a prospective, single-blind, randomized, controlled clinical trial conducted in the NICU and neonatal ward of Boo-Ali, a university-affiliated hospital that is a class III neonatal center in Iran. We obtained informed consent from the parents of all enrolled neonates. The study was approved by the Mazandaran Medical University Research Ethics Committee (code: 89-32) and was registered with the Iranian Registry of Clinical Trials (IRCT). The IRCT code was also IRCT ID: IRCT138812183512N1 (http://www.irct.ir/).

### 2.1. Patients

This study included infants 0–2 months of age of both sexes who were admitted to the NICU or neonatal ward of Boo-Ali Hospital and who were required to undergo urine collection for microbiological studies. The exclusion criteria were a clinical diagnosis of seizures, asphyxia, a decreased level of consciousness, congenital anomalies in the central nervous system, genitourinary and gastrointestinal tracts, abdominal distention, abdominal wall cellulitis, colostomy, inguinal hernia; organomegaly; any hemorrhagic disorder, and the use of any sedative or hypnotic drug.

A random number table was used to assign each eligible neonate to one of two study groups: the “SPA” and “TUC” groups. The randomization was performed by an individual not involved in the other aspects of the study. The assignments were contained in secured, opaque, serially numbered envelopes and opened immediately before urine sampling. To eliminate the effects of sex and weight of patients on the results, the participants in each group were stratified according to sex and weight at the time of admission (less than 1800 g, 1800–2800 g, and over 2800 g).

To calculate the appropriate sample size, we took into consideration the results of a previous study [5] that assessed pain responses using methods very similar to some indices of the PIPP (mean (SD) for brow bulging score 67% (34) for SPA and 42% (38) for TUC), and we estimated that 43 neonates in each group would be required to demonstrate a difference between the groups with a power of 90% and *α* = .05. We also wanted to assess the success rate in each group as a secondary outcome and therefore we powered our study to assess these outcomes as well. We calculated the necessary sample size based on the results of a previous study on the success rates of the suprapubic and catheterization methods 4 (49% for SPA and 77% for TUC). Using a power of 80% and *α* of .05, we estimated that we would require 45 subjects in each group (total 90).

### 2.2. Interventions

A physician who was experienced in the procedures performed all procedures according to standard techniques. In both groups, the procedures were attempted if the infant had not urinated within approximately 30 minutes of feeding. In the SPA group, an anesthetic cream that contained lidocaine and prilocaine was applied locally to the suprapubic area approximately one hour before the procedure. A standard aseptic technique was used. The suprapubic area was cleansed with Betadine solution, and a 22 G needle attached to a 5 mL syringe was inserted at the midline of the suprapubic area, and urine was aspirated [9]. In the TUC group, a standard sterile technique was used. The pelvis and genitalia were cleaned with Betadine solution. We used a sterile 3.5 umbilical catheter or a 5 F feeding tube [5] lubricated with a sterile jelly to catheterize the urethra. We did not use ultrasound or other imaging tools during the SPA or TUC procedures. All the patients were under cardiovascular and oxygen saturation monitoring for approximately 20 minutes before the procedures were started. A procedure was defined as successful when at least 2 mL of urine was obtained. We measured the duration of the urine collection procedure with a stopwatch in seconds.

For all of the patients, we assessed the pain for only the first attempt to collect urine, regardless of the result of the procedure. We assessed pain using the Premature Infant Pain Profile (PIPP), [10] which is a validated method [11, 12] and has been approved by the American Academy of Pediatrics for pain assessment in both term and preterm neonates [8]. The PIPP is based on seven parameters: gestational age, behavioral state, changes in maximum heart rate, changes in minimum arterial oxygen saturation, brow bulging, eye squeezing, and nasolabial furrowing. These indices are individually scored from 0 to 3, and the total score is calculated by summing the scores for these 7 indices. The PIPP score ranges from 0 to the maximum of 18 in term infants and 21 in preterm infants [11].

In addition to monitoring, the neonates' faces and upper bodies were videotaped starting 30 seconds before the start of the procedures and ending 30 seconds after the end of the procedures. We recorded the changes in facial expression, heart rate, and oxygen saturation during the 30 seconds immediately after initiating the procedures. There was no conversation that revealed the type of sampling. Two physicians (one pediatrician and one neonatologist) who were blind to the type of procedure performed watched the videos and independently determined the PIPP score for each patient. The patient was omitted if the scores were different.

The behavioral state was measured by observing the patient for 15 seconds before the procedure was scored according to the PIPP (active/awake, quiet/awake, active/asleep, and quiet/asleep) [10]. The changes in facial expressions were scored by measuring the time that a particular facial change was present in seconds during the first 30 seconds after initiating the procedures. The percentage of the time that the facial change was present was calculated by multiplying this number by 100 and dividing by 30.

The primary outcome was the severity of the pain experienced during these two procedures in neonates as measured by the mean PIPP score. The secondary outcomes were the success rate for urine collection, the rate of contamination of the urine samples, the duration of each procedure, and the rate of complications.

### 2.3. Statistical Analysis

Student's *t*-test was used to analyze continuous variables, and the **χ** ^2^ or Fisher's exact test was used for categorical variables. All analyses were performed using SPSS 13 (SPSS Inc), and a *P* value ≤ 0.05 was considered significant.

## 3. Results

**Figure 1:**
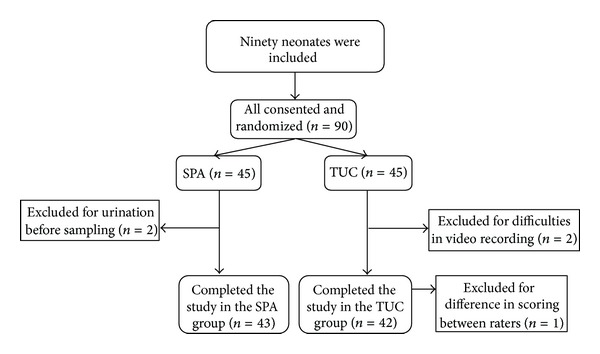
Flow diagram of the study participants.

Ninety infants were recruited, and 45 infants were randomly assigned to each of the SPA and TUC groups. Five patients were excluded, two due to urination before sampling, two due to difficulties with the video recording, and one due to a difference between scores of the two raters. Therefore, a total of 85 neonates, 43 neonates in the SPA group and 42 neonates in the TUC group, completed the study (Figure 1).

Our patients were matched according to sex and weight in the SPA and TUC groups (Table 1). There were no significant differences in the baseline characteristics of the participating neonates between the two groups (Table 1). In our study, seven patients of our male subjects were circumcised (6 in the SPA group and only 1 in the TUC group) (*P* = 0.1). None of the infants were intubated or on oxygen, and none of their faces were covered during the video recording.

The behavioral state scores before starting the procedure did not differ between groups (*P* = 0.4) (Table 2). For all patients, the PIPP scores assigned by the two physicians were similar. There was no significant difference in the PIPP score between the two groups (*P* > 0.05) (Table 2). The physiological responses, although more pronounced for the TUC group, were not significantly different between the two groups (*P* value for heart rate changes = 0 .12 and *P* value for changes in oxygen saturation = 0.06) (Table 2). We analyzed the results of the pain scores separately for boys and girls. The PIPP scores were not different (*P* value = 0.2 for boys and 0.1 for girls). For each sex, we compared the mean PIPP scores for the two methods. For girls, the mean PIPP score for the SPA method was 9.8 ± 4.1, and in the TUC group the mean score was 10.47 ± 2.1 (*P* = 0.5). For boys in the SPA group the mean PIPP score was 10.09 ± 3.39, and for boys in the TUC group, the mean score was 9.08 ± 3.8; the difference between these two scores was not significant (*P* = 0.3).

The success rate did not differ between groups. We were able to obtain an adequate volume of urine from 30 (69.8%) patients in the SPA group and 31 (73.8%) patients in the TUC group (*P* = 0.8). When considering the success rates in the two groups separately for boys and girls, we found that TUC had a significantly higher failure rate in girls than in boys (*P* = 0.01), but the SPA method had similar success rates for both sexes (*P* = 0.6) (Table 3).

No complications occurred during urine sampling in either the SPA or TUC group. There was no need to increase the oxygen support for any of the infants. There was no significant difference in the rate of contamination of the urine cultures between the two groups (*P* > 0.05). There were five positive urine cultures (4 in the SPA group and 1 in the TUC group). Urinary tract infections were diagnosed for the 4 patients in the SPA group with positive urine cultures, but the positive urine culture in the TUC group was considered to be the result of contamination because three different bacteria grew and because a second urine culture for that patient was negative even though no antibiotics had been given.

A comparison of the duration of each procedure showed that the mean time required for SPA group (68.27 ± 3.45 s) was significantly shorter than the time required for TUC (87.11 ± 31.75 s) (*P* = 0.002). A comparison of the duration of the procedures for each sex revealed a significant difference between the two methods in each of the sexes. The TUC method required a longer time, and this difference in duration between the two methods was more significant for girls (in males, the duration of SPA was 66.5 ± 24.3 and the duration of TUC was 80.2 ± 20.9  (*P* = 0.04), and in girls the duration of SPA was 70.1 ± 21.2 and the duration of TUC was 97.2 ± 41.6  (*P* = 0.01)).

## 4. Discussion

Our study did not reveal significant differences in the sensation of pain between SPA and TUC for uncircumcised boys and for girls during the neonatal period. TUC and SPA are both invasive methods [6]. SPA has a low risk of contamination [1, 3, 4, 13] but must be performed by experienced physician. In addition, different success rates have been reported for this procedure [2, 5]. TUC also has an acceptable sensitivity and specificity [3]. Although TUC is performed by nurses in some centers, this procedure also requires sufficient expertise and knowledge. TUC may be difficult and painful, especially in girls, small infants, and uncircumcised boys [1, 5, 6].

Experiencing severe or prolonged pain may increase neonatal morbidity [14] and is associated with changes in physiological parameters [8]. Therefore, the least painful medical interventions should be selected for neonates [5, 8]. There have been few studies (to our knowledge, only two) conducted to compare pain in neonates during SPA and TUC, and, to the best of our knowledge, the present study is the first on this subject for uncircumcised boys. In small uncircumcised male infants, the retraction of the prepuce can be difficult and painful, and therefore the TUC method can be more painful. In our study, we used the PIPP to assess pain. This scoring method is based on a combination of behavioral indicators and changes in physiological parameters in response to pain [12], which shows the value of this scale for the assessment of neonatal pain. In our study, the PIPP score was determined independently by two physicians to increase the reliability of the pain scoring results.

The changes in heart rate were not different significantly between the two groups. The greater decrease in arterial oxygen saturation in the TUC group (−2.5 in SPA and −3.2 in TUC), although not statistically significant (*P* = 0.06), may be indicative of the difficulty of TUC.

The two other studies on the pain experienced by neonates during these two procedures concluded that the SPA method is more painful than TUC [1, 5]. In Kozer's study, although the VAS and DAN scores were higher for SPA method, the duration of crying did not differ between the two groups [1]. The duration of crying is often used to assess the level of pain experienced by infants [1, 12, 15]. The patients included in our study were smaller than the patients in Kozer's study. In addition, all boys in our study were uncircumcised, but in Kozer's study all the boys were circumcised. Finding the urethral meatus by retraction of the prepuce may be more difficult in smaller infants and in uncircumcised boys, thus making TUC more difficult [1, 6, 7].

The DAN and VAS scoring systems are valid tools for the assessment of pain in neonates but do not measure the changes in physiological parameters. In addition, in Kozer's study the measuring of the VAS scores [1, 16] was not blinded, and (as previously stated by other authors [1]) therefore these scores may reflect only the impression that SPA is a more painful procedure. In the other study, which was performed with preterm neonates by El-Naggar et al. [5], the researchers found that SPA was significantly more painful [5], which is contrary to our results. There are some differences between these two studies that may explain the different results. The gestational age of the patients in El-Naggar's study was much lower (a mean gestational age of approximately 27). In our study, there was only one patient with a gestational age less than 32 weeks, and more than 90 percent of patients in El-Naggar's study were ventilated, which could limit the assessment of facial grimacing. Similar to our findings, their assessment of changes in the heart rate and oxygen saturation did not show any difference between TUC and SPA. Although they may be less sensitive than behavioral parameters [5], changes in physiological parameters are important and are used in many validated pain tools [8, 12]. In addition, in preterm infants, maintaining the stability of physiological parameters such as the heart rate and oxygen saturation is very important to prevent morbidities such as intraventricular hemorrhages and periventricular leukomalacia [17].

Oswald et al. [18] compared the level of pain between SPA and TUC during voiding cystourethrography. They showed that there was an overall statistically significant lower level of pain in the SPA group, in contrast to the results of Kozer's study [1]. They also reported that TUC was significantly more painful in girls, similar to our results. Because of the older age of the patients, the trend of increasing pain in the TUC group with increasing age found in this study could not be confirmed for younger infants, whose smaller anatomy may lead to greater difficulty in performing TUC.

Our success rates were obtained even though ultrasound guidance was not used during the SPA procedures. Most of our participating neonates were icteric patients and were thus generally well hydrated. This adequate hydration might have increased the success rate in our SPA group.

The significantly higher failure rate (*P* = 0.01) and longer duration for the TUC method in girls found in this study were similar to the results of a previous study, 6 indicating that TUC is more difficult in girls.

The incidence of UTIs in our study (2.3%) was comparable with that found in other studies [1, 3, 5, 9, 13] and the low rate of contamination of the urine samples is similar to the rates found in other studies [1, 4–6]. These results indicate that both the TUC and SPA methods may be reliable for sterile urine collection from neonates.

All the procedures in this study were performed by one person to ensure that the technique was consistent, but having the procedures performed by only one person may limit the generalizability of the findings to every level of clinical practice and skill. However, we believe that, in any clinical situation in which it is rational to speak about the level of pain associated with a procedure, the procedure should be performed by a trained professional and in a standard manner. The calculation of the durations of the procedures might be subject to bias because it depended on an (unblinded) individual using a stopwatch. This potential bias is also a limitation of this study.

## 5. Conclusion

The conclusions regarding the secondary outcomes of the subgroups are tentative due to the small sample size. Our study showed that there is no difference in the level of pain experienced during SPA and TUC in uncircumcised boys and in girls during the neonatal period, and both of the methods can be used successfully for the collection of sterile urine samples. However, the probability of greater difficulties with TUC in girls and uncircumcised boys must be taken into account.

## Figures and Tables

**Table 1:** Baseline characteristics of the participating neonates.

Procedure	SPA (*n* = 43)	TUC (*n* = 42)	*P*
Weight			0.7
<1800 g	1 (2.3%)	2 (4.8%)	
1800–2800 g	8 (18.6%)	9 (21.4%)	
>2800 g	34 (79.1%)	31 (73.8%)	
Gestational age			0.5
28–31 6/7 wk	0 (0%)	1 (2.3%)	
32–35 6/7 wk	9 (20.9%)	7 (16.6%)	
≥36 wk	34 (79.1%)	34 (80.9%)	
Sex			0.4
Male	22 (51.2%)	25 (59.5%)	
Female	21 (48.8%)	17 (40.5%)	
Gestational age, mean (SD), wk	37.3 (2.1)	37.2 (2.6)	0.9
Chronological age, mean (SD), d	13.9 (16.4)	11.5 (12.9)	0.9
Weight (at admission), mean (SD), g	3296.5 (903.5)	3056.9 (628.4)	0.16
Diagnosis			
Icter, *n* (%)	30 (69.8%)	28 (68.3%)	
Sepsis, *n* (%)	6 (14%)	5 (12.2%)	0.9
Others, *n* (%)	7 (16.3%)	8 (19.5%)	
Circumcision of males	37 (86%)	41 (97.6%)	0.1
No, *n* (%)			
Yes, *n* (%)	6 (14%)	1 (2.4%)	

**Table 2:** Pain assessments in neonates during SPA and TUC using the PIPP.

Pain assessments	SPA (*n* = 43)	TUC (*n* = 42)	*P*
Behavioral state			0.4
Active, *n* (%)	32 (74.4%)	28 (66.7%)	
Quiet awake, *n* (%)	3 (7%)	2 (4.8%)	
Active asleep, *n* (%)	3 (7%)	8 (19%)	
Quiet asleep, *n* (%)	5 (11.6%)	4 (9.5%)	
Brow Bulge Score, mean (SD)	2.2 (1.1)	2.1 (1.1)	0.7
Eye Squeeze Score, mean (SD)	2.1 (1.1)	2.1 (1.1)	0.8
Nasolabial Furrow Score, mean (SD)	2.9 (4.6)	2.1 (1.1)	0.2
Max. HR change, beats per min, mean (SD)	22.6 (18.8)	16.6 (16.5)	0.12
O2 sat. change, mean (SD)	−2.5 (4.3)	−3.2 (5)	0.06
PIPP score, mean (SD)	9.9 (3.7)	9.6 (3.2)	0.6

**Table 3:** Comparison of the success rates of SPA and TUC for male and female neonates.

Success	Successful	Failure	*P*
SPA			0.6
Male, *n* (%)	16 (72.7)	6 (27.3)	
Female, *n* (%)	14 (66.7)	7 (33.3)	
TUC			0.01
Male, *n* (%)	22 (88)	3 (12)	
Female, *n* (%)	9 (52.9)	8 (47.1)	
